# Assessing Active and Passive Glenohumeral Rotational Deficits in Professional Tennis Players: Use of Normative Values at 90° and 45° of Abduction to Make Decisions in Injury-Prevention Programs

**DOI:** 10.3390/sports13010001

**Published:** 2024-12-24

**Authors:** Maite Terré, Juliette Tlaiye, Monica Solana-Tramunt

**Affiliations:** Facultat de Psicologia Ciències de l’Educació i l’Esport (FPCEE) Blanquerna, Universitat Ramon Llull, 08022 Barcelona, Spain; maitetp@blanquerna.url.edu (M.T.); juliettetp@blanquerna.url.edu (J.T.)

**Keywords:** shoulder, glenohumeral internal rotation deficit (GIRD), tennis players, range of motion, glenohumeral external rotation deficit (GERD), injury prevention, overhead athletes, elite athletes

## Abstract

(1) Background: Glenohumeral internal and external rotational range-of-movement deficits (GIRDs and GERDs) are some of the primary outcomes used to determine the risk of injury in overhead athletes, such as tennis players. Nevertheless, the current testing position does not consider the fact that most tennis actions are repeated at 45° of abduction, and actively. The aim of this study was to establish normative values of pathological GIRDs and GERDs in tennis players and to provide normative values for both the passive and active rotational range of motion of the glenohumeral joint at 90° and 45° of abduction. (2) Methods: Forty-three tennis players voluntarily participated in this study (19.1 ± 2.75 years). The dominant and non-dominant total glenohumeral rotational range of motion (TRROM), external rotation (ER), and internal rotation (IR) at 90° and 45° under active and passive conditions were evaluated. The GIRD and GERD were calculated in both positions and under both conditions. (3) Results: There were significant differences in all of the passive measurements between the 45° and 90° testing positions. The ER and TRROM at 90° and 45° showed significant differences under both passive and active conditions and on the dominant and non-dominant sides. Actively, there were no significant differences in the IR or TRROM for either the dominant or non-dominant side at 90° or 45°. (4) Conclusions: It is necessary to evaluate ER under the same conditions at 90° or 45°. Practitioners should consider assessing the ER for the angle at which most actions are repeated in tennis (45°) as a method to monitor GERDs. Evaluating GERDs in asymptomatic tennis players could help avoid future biomechanical and GIRD problems. Both GIRDs and GERDs should be considered as a percentage of the athlete’s own deficit in IR or ER, instead of referencing specific degrees that have been observed in baseball pitchers.

## 1. Introduction

Tennis is considered one of the sports that places a high demand on the glenohumeral joint [1]. The technical requirements in tennis require the development of kinetic chains from the ground to the racket to generate force when hitting the ball. In this kinetic chain, the glenohumeral joint of the dominant shoulder is crucial during the initial phase, acceleration, and final phases of a tennis stroke. An enhanced rotational range in the dominant shoulder can provide advantages for tennis players [1].

The static and dynamic mobility of the shoulder complex depends on the synchronized action of the surrounding muscles and the elastic properties of the soft tissues passing through its joints [2,3,4,5]. The demands on these structures are significantly increased in overhead sports such as tennis. While each overhead sport has distinct characteristics, they share similar movement patterns and expose the upper limb joints to repetitive overhead actions [6,7,8].

The repetitive strokes that tennis players must make in every practice and match, with a lot of power and acceleration, can increase the stiffness of the muscles and the posterior structures of the shoulder and compromise the IR of the glenohumeral joint in the dominant limb compared to the non-dominant limb, causing asymmetries in both strength and joint mobility [9,10,11]. The numerous serves made throughout a tennis match can cause recurrent microtrauma and may lead to physiological adaptations of the joint as well as the joint’s surrounding soft tissue [12]. Permanent tightness in the posterior rotator cuff and posterior deltoid leads to alterations in the scapular and humeral kinematics in tennis athletes [13]. These biomechanical adaptations often result in decreased glenohumeral internal rotation (GIRDs) combined with a decreased total glenohumeral rotational range of motion (TRROM) of the dominant compared to the non-dominant limb [13]. In fact, repetitive overhead movements in sports are considered one of the risk factors for developing GIRDs. Thus, appropriate prevention or early intervention may be necessary for all overhead sports players [4,8].

A GIRD is defined as a decrease in the glenohumeral internal rotation range of the dominant shoulder compared with the non-dominant shoulder [14,15]. GIRDs are related to an increased risk of a shoulder injury, and consequently, they affect the continuity of the practice and performance of tennis players [12,13]. Most studies regarding the shoulders of overhead athletes have focused on baseball pitchers or other throwing athletes. Nevertheless, the technical requirements and the injury etiology in pitchers are very different from those in tennis players [16,17].

The current literature differentiates between non-pathological and pathological GIRDs. A pathological GIRD is defined as a loss of IR in the dominant shoulder greater than 18°–20° compared to the non-dominant shoulder [15,18,19]. In addition, other authors have defined a pathological GIRD as the loss of glenohumeral IR degrees together with a loss of TRROM greater than five degrees [14]. Nevertheless, these descriptions have only been defined in pitchers [14,18,19,20].

The shoulder is one of the most commonly injured joints in tennis players [9,13,21,22]. The dominant side is affected by 15.9% of all overuse injuries in junior tennis athletes [22,23]. Younger tennis players show chronic overuse injuries and an increased risk of acute injuries due to the repetitive stress on the glenohumeral joint in biological maturate phases [8,13,21]. Aging has an impact on reducing the IR and, therefore, the glenohumeral TRROM, on the side most often used to develop strength [8,12,24]. Changes in rotational shoulder ROM are more common after years of tennis practice [13,16].

In tennis, a balance between the internal and external glenohumeral muscles is recommended. The strength of the external rotators should be at least two-thirds of the strength of the internal rotators [11]. In fact, muscle asymmetries have been demonstrated to have an impact on the dominant rotation ROM and cause a higher injury risk or shoulder pain in overhead athletes [11].

To obtain accurate and reliable results, researchers have used a goniometer as a tool to calculate the range of motion of a joint. A goniometer is the most widely used tool to calculate ROM passively, as it is easy to use and inexpensive and has proven its validity [25]. When the aim is to assess the passive glenohumeral joint motion, clinicians agree that it is important to stabilize the rest of the joints of the scapulohumeral complex, especially the scapula, to ensure the isolation of the glenohumeral movement [17]. Moreover, previous studies have agreed that the most reliable method for assessing the passive glenohumeral rotational range of motion (ROM) is scapular stabilization [17,25]. Nevertheless, the need to use both hands when operating a goniometer makes it challenging to stabilize the trunk and scapula, increasing the chances of measurement errors [4,26,27,28]. The precision in measurements of the glenohumeral mobility with a goniometer varies depending on the method chosen, as well as the practice and experience of the evaluator, so interobserver reliability is low [28]. The growing popularity of commercially available motion-tracking devices has led to the increased use of wearable motion-capture systems to obtain easy, reliable, and valid measurements of ROM [28,29,30,31]. In this regard, inertial measurement units (IMUs) have gained widespread use because of their ease of use, cost-effectiveness, and portability [28,29,31,32].

Shoulder ROM assessments in tennis players have been recommended because they are essential for evaluating muscular conditions [27], maintaining healthy shoulder rotation, and reducing the risk of a shoulder injury [1,8,13,33]. Since healthy glenohumeral rotation may be beneficial to the biomechanics of the tennis stroke and the prevention of shoulder injuries, junior tennis players in particular should receive periodic surveillance of their shoulder ROM [1,8]. Previous studies have agreed with the importance of analyzing the joint in the position that most closely resembles specific tennis movements to obtain more accurate information on the functionality of the joint [11,34]. Furthermore, regarding the side-to-side asymmetries, the dominant shoulder demonstrates adaptations of shoulder ROM, with a reduction in the IR and TRROM and an increase in the ER when compared with the non-dominant side [11].

The current tests used to assess the glenohumeral rotational range of motion were developed at 90° of glenohumeral abduction [1,11,13,16,23,24,35]. However, most of the powerful actions in tennis are developed at 45° of abduction [23,24]. Moreover, to our knowledge, there is a lack of studies that have considered the possible differences in the ER or the GERDs and GIRDs actively and passively at 90° and 45° of abduction. Understanding the tennis-specific adaptations of the shoulder complex could help tennis players, coaches, athletic trainers, and clinicians to design optimal exercise protocols. Therefore, the aim of this study was to establish baseline models of pathological GIRDs and GERDs in tennis players, in order to determine the differences between the passive and active rotational range of motion of the glenohumeral joint at 90° and 45° of abduction. We hypothesized that the glenohumeral rotational range of movement would be significantly different when it is developed at 90 vs. 45° of abduction, and that the values for the IR, ER, and TRROM would be different between the dominant and non-dominant sides at 45° and 90°.

## 2. Materials and Methods

### 2.1. Study Population

A sample of forty-three tennis players, both males and females (age, 19.1 ± 2.75 years; height, 1.77 ± 0.08 m; body mass, 69.7 ± 10.3 kg; IMC, 22.2 ± 2.33 kg/m^2^), was recruited from five different high-performance Spanish tennis academies and clubs. Four players had an Association of Tennis Professionals (ATP) ranking (17.8 ± 16.8 ATP ranking), twenty-nine players had an International Tennis Federation (ITF) junior ranking (22.6 ± 12.1 ITF junior ranking), and ten players had a national ranking in their country (21.9 ± 13.3). All of the tennis players were healthy and competing at the time of the trial (March–April). Other characteristics regarding their demographics, shoulder pain, dominance, and volume of training are shown in Table 1.

All of the participants met the inclusion criteria (Table 2) and were informed, both in writing and verbally, about the procedures of the study prior to the assessment day. After receiving detailed information, each participant provided signed informed consent according to the latest version of the World Medical Association’s Declaration of Helsinki (2013) [36].

### 2.2. Data Collection

All data collections were performed during the pre-season months of December (2023) and February (2024). All of the measurements were carried out in the usual training facilities. Before the evaluation day, the researchers contacted the head coach to schedule the time of each measurement in order to avoid interrupting tennis practice and improve the ecological validity of the study. Upon the arrival of each participant, the purpose of the study and the examination technique were explained and a demonstration in each arm was provided, and those who agreed to participate provided signed informed consent. None of the tennis players who met the inclusion criteria refused to participate.

Before taking the measurements, the participants answered a questionnaire to obtain additional information about their sports practice, which side was their dominant side (or which of their upper limbs they thought they had the most strength with), and their history of shoulder pain, among other questions. In this questionnaire, they also provided descriptive measures, such as their year of birth, body mass, and height (Table 1).

**Figure 1:**
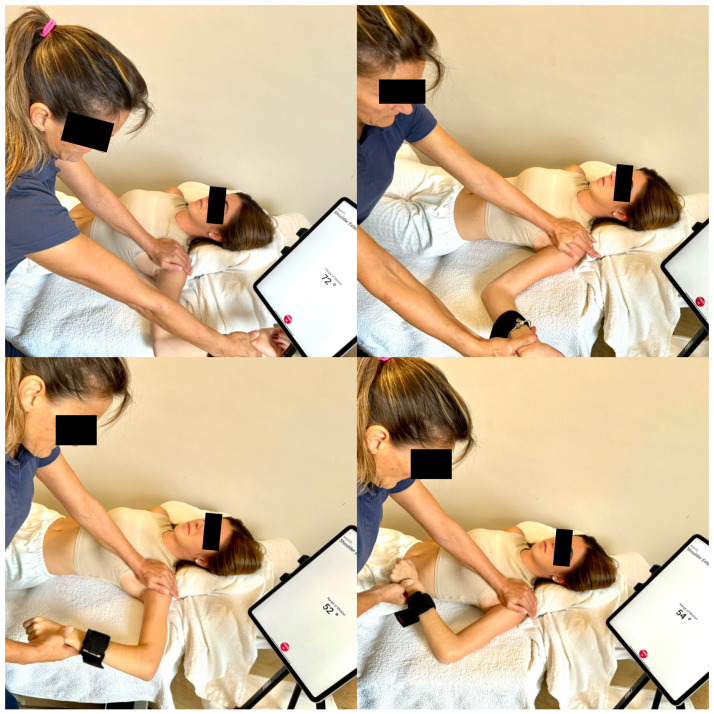
Passive assessment settings at 45° and 90° with Output Capture IMU.

All of the tennis players were examined once, at rest and without having practiced in the previous hour. An experienced researcher performed all the measurements with an IMU Output Capture sensor (Output V2 Unit, Ireland). The sensor was attached to the participant’s forearm with a self-adhering strap at 10 cm to the lateral epicondyle. The calibration of the sensor was set prior to testing according to the app instructions. All of the measurements were shown wirelessly on the tablet screen and subsequently transferred to the output hub to extract the IR and ER data (Figure 1).

### 2.3. Passive Assessment in Supine Position

All of the measurements were developed by a world-level sport physiotherapist and osteopath with 30 years of experience working with elite athletes.

The subjects laid in a supine position with the tested arm at 90° or 45° of glenohumeral abduction. To ensure the exact abduction of each position, tape was attached to the massage table at 90° and 45° from the mid-longitudinal axis of the table. The participant’s humerus was placed on the tape marks for each position. The forearm position was perpendicular to the supporting surface (with the elbow at 90° of flexion) and at 0° of supination and pronation, so that the palm faced inferiorly. The elbow was outside the plane of support, exactly the width of each participant’s hand to avoid obstacles to the glenohumeral ER. The scapular movement was avoided using overpressure on the coracoid process. No underarm support was needed because the 10 and 15° of difference between the humerus plane and the scapula disappeared with the light non-painful overpressure, as the scapula sunk softly into the table’s foam. The experienced researcher controlled the maintenance of the glenohumeral joint at the desired position (90° or 45° of abduction), pushing lightly from the distal part of the forearm to keep the humerus within the desired amplitudes. Towards the end of the ROM, the clavicle and the acromion and coracoid processes of the scapula were stabilized to avoid the anterior tilting of the scapula [17,35]. From this starting position, the researcher held the participant’s clavicle and scapula against the table to stabilize the scapula while rotating the humerus in the glenohumeral joint to produce the maximum passive ER and IR (Figure 1). For both the 90° and 45° measurements, glenohumeral rotation began with the neutral position and ended when firm non-painful resistance to passive rotation was achieved. The sensor automatically recorded the degrees of rotation obtained when the position remained static for 2 s. Special attention was paid to limiting the movement to pure glenohumeral rotation and minimizing compensatory movements of the scapula–thoracic region, especially during the IR assessment [11,35].

### 2.4. Active Assessment in Seated Position

To extract active glenohumeral rotation data, the participants were asked to sit on the massage table with the glenohumeral joint at 90° or 45° of abduction and with the elbow at 90° of flexion. The examiner remained next to the tennis players to ensure that the evaluation position was maintained throughout the entire rotation path without any type of postural compensation occurring that could bias the degrees of rotation.

Three sets of each movement were performed on each side, first at 90° of abduction and then at 45°. When the tennis players reached the end of the movement and stayed in that position for two seconds, the IMU automatically registered the achieved degrees and sent the data to the application.

The order of the evaluation was passive testing (supine) first, as follows: IR and ER, 90° on the left side; IR and ER, 45° on the left side; IR and ER, 90° on the right side; and IR and ER, 45° on the right side. The same order was followed to assess the active seated glenohumeral rotation. The evaluation times for each player were 5 min to answer the questionnaire and 15 min for all of the ROM assessments. There were no complaints during the measurements.

The TRROM was calculated as the sum of the IR and ER for each side. The glenohumeral ER deficit (GERD) was calculated as the difference between the dominant ER and the non-dominant ER degrees. We classified 3 different kinds of GIRDs: Type I was calculated as the absolute difference between the dominant and the non-dominant IR and ER degrees. Type II was calculated as the percentage (%) of difference between the dominant and non-dominant TRROM. Type III was defined as the percentage (%) of difference between the dominant and non-dominant IR and ER [25,37].

Moreover, we defined 3 types of pathological GIRDs: Type I was an IR deficit > 18° with a loss in the TRROM of >5° when compared to the contralateral shoulder [13,14,18]. Type II occurred when the difference between the TRROMs of each shoulder was >10% [19]. Type III occurred when the difference between the dominant and non-dominant IR was >20%.

We defined a pathological GERD as occurring when the difference between the dominant and non-dominant ER was >20%.

### 2.5. Statistical Analysis

Descriptive statistics (means, standard deviations, and percentages) were calculated for each of the demographic and descriptive outcomes (Table 1). The normality of the data distribution was verified using the Shapiro–Wilk test.

A paired *t*-test was used to analyze the differences between all of the outcomes (IR, ER, TRROM, GIRD I, GIRD II, GIRD III, and GERD) at 90° vs. 45° for active and passive conditions, and for comparisons between the dominant and non-dominant limb. The statistical significance was set at *p* < 0.05.

Cohen’s d was used to determine the effect size (ES) for all of the comparisons. The effect size (ƞ^2^) was calculated and interpreted as follows: ƞ^2^ < 0.02, no effect; ƞ^2^ = 0.2–0.49, small effect; ƞ^2^ = 0.50–0.70, moderate effect; and ƞ^2^ > 0.80, large effect.

All of the analyses were performed using JAMOVI, version 2.5.4. The results are shown as the mean (SD) and N (%), and the significance level was set at *p* < 0.05 when the ES’s 95% confidence interval limits did not cross the zero value.

## 3. Results

### 3.1. Dominant and Non-Dominant Comparisons at 90° and 45°

**Figure 2:**
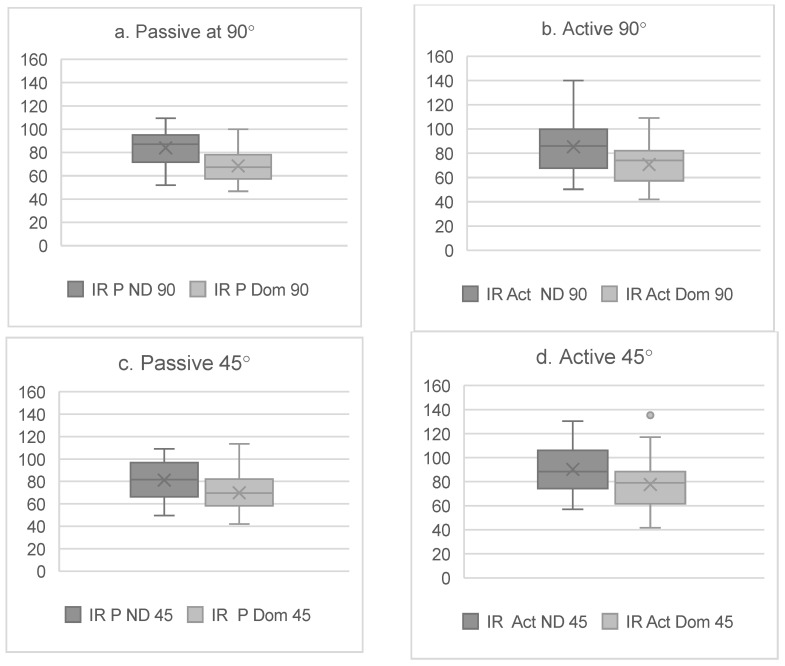
(**a**) IR for dominant (Dom) and non-dominant (ND) sides for passive condition at 90°. (**b**) IR for dominant (Dom) and non-dominant (ND) sides for active condition at 90°. (**c**) IR for dominant (Dom) and non-dominant (ND) sides for passive condition at 45°. (**d**) IR for dominant (Dom) and non-dominant (ND) sides for active condition at 45°.

**Figure 3:**
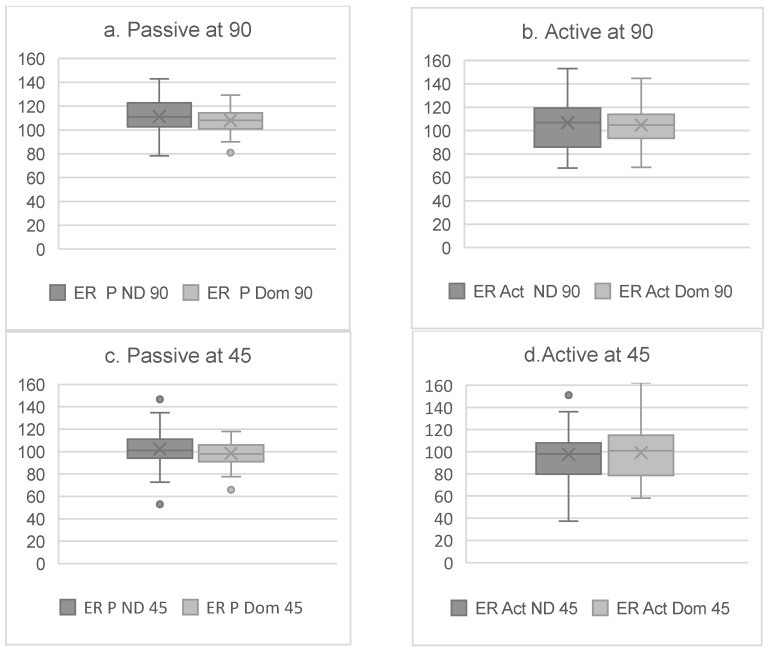
(**a**) ER for dominant (Dom) and non-dominant (ND) sides for passive condition at 90°. (**b**) ER for dominant (Dom) and non-dominant (ND) sides for active condition at 90°. (**c**) ER for dominant (Dom) and non-dominant (ND) sides for passive condition at 45°. (**d**) ER for dominant (Dom) and non-dominant (ND) sides for active condition at 45°.

We found significant differences (*p* < 0.001) between the dominant and non-dominant sides for the IR, ER, and TRROM degrees in all test positions and under all conditions (Figure 2 and Figure 3).

### 3.2. Differences Among Measurements at 90° and 45°

The results showed significant differences between the 90° and 45° scores for the passive ER of both the dominant and non-dominant sides, and for the ER of the non-dominant side only when assessed actively. There were significant differences in the TRROM assessed under passive conditions for both the dominant and non-dominant sides at 90° and 45°. The IR results were only significantly different for the dominant side assessed under active conditions at 90° and 45° (Table 3).

### 3.3. Descriptive for GIRD and GERD at 90° and 45°

Results for different types of GIRD and GERD assessed at 90° and 45° are displayed in Table 4.

## 4. Discussion

To the best of our knowledge, this study presents, for the first time, reference data for the glenohumeral rotation at 45° and 90° actively and passively, and different types of GIRDs and GERDs in tennis players. This highlights the need for reference values that are more specific than most of the evaluations used in previous studies. It has been reported that specific information regarding the bilateral comparisons of a normal range of motion in healthy, uninjured athletes is of importance, as these comparisons are often used in clinical decisions regarding the extent and magnitude of range-of-motion loss and subsequent strategies to regain motion after an injury or surgery [14,16,38].

The results in the present study showed significant differences for all rotational movements between the dominant and non-dominant sides (Figure 2 and Figure 3). In line with previous research on tennis players, the IR was significantly lower on the dominant side [13,23,35,37,39,40]. In contrast, the ER degrees were not greater on the dominant side (Figure 3). These differences could be caused by the absence of pain or injuries in the glenohumeral joint of the participants in the present study, as one of the inclusion criteria was not suffering any pain or injuries within the two weeks prior to the testing day. Nevertheless, 67.4% of the tennis players in the present study reported having a shoulder injury story and 25.5% reported shoulder pain during practice. Another possible explanation is that 86% of the participants were involved in a shoulder-injury-prevention program, which could have enhanced the strength and stability of the shoulder complex and reduced ER asymmetries [41,42].

The primary aim of this study was to add information about IR in the range of movement that often occurs in tennis players, at 45° of glenohumeral abduction under active conditions. Therefore, we compared the results of the IR of the dominant and non-dominant shoulders developed at 45° and 90° of abduction under active and passive conditions. When comparing the results for the passive condition at 90°, we observed that the IR values in the present study were higher than those in previous studies [23]. A possible explanation for these results is the fact that we did not use a goniometer and that there was the possibility of pushing the glenohumeral joint harder than other researchers who needed to move the goniometer arms while the participants moved their forearm, causing a limitation in the range of movement. In the present study, we added an extra 10° in comparison with previous studies [23] by bringing the glenohumeral joint to the maximal passive internal or external position without causing any pain. Another possible explanation is that, in previous similar studies, the subjects had shoulder pain or reported an injury when they were assessed, and these could have caused IR restrictions [23,35]. Unfortunately, to our knowledge, there are no previous studies that can be used to compare the 45° rotational ranges of movement developed in tennis players.

There were significant differences between the IR developed at 45° or at 90°. Our results showed that the IR values at 45° of abduction were lower than those at 90°. This difference can be attributed to several biomechanical factors. In the 45° position, the posterior shoulder capsule and the posterior deltoid are in a less restrictive position. This allows the posterior rotator cuff to be more stretched, which may limit its ability to internally rotate [10,24]. In contrast, at 90° of abduction, the tension on these structures decreases, which may facilitate greater internal rotation due to a better alignment and less movement restriction [23,35,39]. In addition, the 90° abduction position can optimize the shoulder biomechanics, allowing for the greater activation of internal rotator muscles, such as the subscapularis, which contributes to an increase in IR values [18,39]. Therefore, it is critical to consider the shoulder position when assessing internal rotation, as this can have significant implications in the rehabilitation and training of athletes.

There were no significant differences when the internal rotation was evaluated actively at 90° and 45°. A possible reason could be that, when the assessment was performed actively, the tennis players compensated for the range of movement with the internal rotation musculature, which is well strengthened in professional tennis players, and the force of gravity. Moreover, when we assessed internal rotation passively, the shoulder forward thrust was blocked, and in the seated active assessment, this block was not possible.

When comparing the differences in the TRROM of the glenohumeral joint at 90° and 45° of abduction, we found significant differences. These variations can be attributed to several biomechanical and physiological factors affecting joint mechanics. First, the position of the arm influences the tension and length of the muscles and ligamentous structures surrounding the glenohumeral joint. At 90°, muscles such as the supraspinatus and infraspinatus are in a different position of contraction and stretch compared to at 45°, which can alter the TRROM [9,33,39]. Furthermore, at 90°, the effect of gravity is greater, which may restrict passive movement due to a greater load on the stabilizing structures [9,43]. At 45°, the articular surfaces may align more favorably, allowing for a greater passive range of motion. This variation in congruency can result in differences in the distribution of forces within the joint, directly affecting the range of motion of the glenohumeral joint [24,43].

Identifying these differences in passive TRROM between 90° and 45° is crucial in clinical assessments and rehabilitation. A thorough understanding of how these positions affect shoulder mobility will allow clinicians to develop more accurate and personalized assessment protocols for recovery from shoulder injuries, because this means that passively measuring the ROM of the glenohumeral joint at 90° will give very different data to those that can be developed at 45°. However, it seems that, when the amplitude is actively performed, there are no significant differences or important effect measurements in either the dominant or non-dominant side at 90° and 45°.

The asymmetric rotational ROM is considered a specific adaptation in tennis players caused by the high repetitive loading forces generated by strokes, mainly the serve and groundstrokes. Each sport technique involves different joint amplitudes and different working angles of the muscles around the joint. When a joint changes its physiological neutral position, the efficiency of all of the muscles that pass through it changes, and the working angle changes the torque and, with it, the efficiency of the muscles passing through the joint [1,24].

There were significant differences in external rotation at 90° and 45° for the dominant and non-dominant sides. An assessment of the ER at 90° and 45° showed significant differences between the dominant and non-dominant sides under both active and passive conditions. We found a lower ER at 45° for both the active and passive conditions. It is important to note that, for active actions at 45°, the supraspinatus is completely activated to develop the first 30° of the abduction, and the posterior deltoid starts to contract to perform the rest of the abduction until 45°, when it starts its participation in the ER movement of the humerus. Therefore, these statistically significant influences can be explained by the position of the arm, because there is an influence of the available motion of the glenohumeral joint. At 90°, the external rotator muscles, such as the infraspinatus and teres minor, are more active and stiffer. This may restrict the ER compared to the position at 45°, where the muscle mechanics allow for a greater freedom of movement [23,39,40]. In addition, at 90°, scapular stabilization plays a crucial role; any dysfunction in this area can result in a significant decrease in the range of motion [39]. Moreover, it has been reported that the rotational velocities involved in tennis, particularly during serving, can be even higher than those observed in baseball batting. This suggests that the stress and fatigue on the posterior capsulolabral complex, rotator cuff, and posterior deltoid could lead to a reduced ER under active conditions and in situations where these movements are more frequently repeated in tennis. Additionally, this technical requirement in tennis has reportedly increased the risk of posterior shoulder instability (PSI) for tennis players [43]. Thus, we must consider both active and passive conditions to obtain a complete evaluation of tennis players. Additionally, we consider it important to know the ER for the angle at which most tennis actions are repeated (at 45°), as the glenohumeral ER restrictions at 45° generate biomechanical problems around the waist that, if not detected in time, could cause future GIRDs and/or symptomatic injuries in tennis players [35,37,40,43].

We defined a pathological GIRD as occurring when the difference between the IR of the dominant and non-dominant shoulders is greater than 20% or when there is a difference of 10% in the side-to-side TRROM. Previous studies have stated that only a 5° difference in the TRROM or IR differences of 18–20° increase the risk of injury in baseball pitchers. However, we found that these percentages corresponded to greater differences in the absolute degrees than those described for pitchers. Moreover, several authors have stated that GIRDs could develop after long periods of a restriction in the glenohumeral external rotation range of movement (GERD). Nevertheless, a few studies have focused on describing the problems that GERDs could cause to overhead athletes [44,45,46]. The results showed that, in asymptomatic tennis players, an increased workload and stiffness in the internal rotators of the glenohumeral joint resulted in a restriction of the ER. Stiffness in the anterior muscles pulls the major tubercle of the humerus to IR and, therefore, the humeral head experiences posterior displacement, opposite to the biomechanical throwing technique reported for pitchers [4,16,20,25]. This change in the neutral position of the humeral head reduces the torque of the posterior rotator cuff, increasing the work that it must perform to externally rotate the humerus during the glenohumeral abduction. Thus, the external rotation produced is not enough to avoid the collision between the major tubercle of the humerus and the acromion, and the impingement of the subacromial space is repeated in every overhead action of these athletes. With all of this, the repetitive impingement can lead to the development of a pathology [47,48]. For this reason, it is very important to take GERDs into account in the evaluation of tennis players.

The variation in passive mobility between the 90° and 45° angles can be explained through the concept of muscle tension and joint coaptation. At 45°, the alignment of joint structures may be more favorable, allowing a greater range of motion without the restrictions imposed by muscle tension [9,22,33,40]. This suggests that measurements in different positions may provide complementary information on joint health and muscle function.

Depending on whether the evaluation is performed at 90° or 45° and under active or passive conditions, we can see how these factors can influence the precision, reproducibility, and accuracy of the measurement; however, this may not be related to glenohumeral motion. It is inappropriate to assume that all of these variables are the same on both sides, since the motion of the elbow, scapular pronation, and scapular supination are manifestly different on the dominant and non-dominant sides [39]. A previous study assessed the passive glenohumeral IR and ER of elite tennis players in a seated position and found a decreased dominant IR and an increased dominant ER [40]. These results are similar to those of studies that have assessed the passive glenohumeral ROM in a supine position [23,33,39]. Nevertheless, there is still a lack of studies comparing different angles and conditions for evaluating the glenohumeral ROM in tennis players.

The absolute IR degrees at 45° and 90° of abduction were lower on the dominant side than on the non-dominant side under both active and passive conditions. However, the active and passive ER at 45° and 90° were greater on the non-dominant side than on the dominant side. Our results are mostly in line with previous research, except for the greater ER on the non-dominant side [13,21,23,35,37,39]. This discrepancy could be associated with the fact that most of the previous studies in tennis players were developed on injured or painful shoulders, and in the present study, the participants stated being free of shoulder pain. Since it is well known that, in a painful tennis shoulder, the increase in the ER on the dominant side is an adaptation to the restrictions in the IR on the painful dominant side [16,24,40], our findings suggest that the detection of a restriction in the non-dominant shoulder could be a sign of a possible biomechanical problem that may trigger future problems in the dominant limb.

The association between GIRDs and an increased risk of shoulder injuries in tennis athletes has been frequently reported. Moreover, an increased side-to-side shoulder IR difference and a decrease in the ER/IR ratio have been reported to have a significant relationship with the years of tennis practice [13]. The fundamental reason behind these differences lies in the functional adaptation of the dominant shoulder. Athletes and individuals who perform unilateral movements develop greater strength and neuromuscular control in the dominant arm, which can lead to an imbalance in flexibility and range of motion. In this regard, the repeated use of the dominant arm in activities requiring strength and precision may result in greater stiffness of the muscles and connective tissues compared to the non-dominant side, which may be freer to develop an optimal range of motion [23,35,40].

Although previous studies imply a relationship between upper extremity injuries and GIRDs in overhead athletes, this is the first study to consider GERDs as a method for detecting possible GIRDs in tennis players. Most of the literature establishing criteria for addressing painful shoulders in athletes has investigated the biomechanics and pathology of baseball pitchers [8,16,49]. However, the technical requirements and the injury etiology in pitchers are very different than those of other overhead athletes such as tennis players. To the best of our knowledge, there is a lack of studies about the shoulder injury etiology for specific technical movements in tennis. For this reason, this study could be useful for determining a specific normative value in tennis players at 45° of abduction.

Most previous studies have considered a GIRD as an index for injury prevention, but there is still a lack of knowledge about normative values for GERDs. In tennis players, before we are able to observe a GIRD, there is a decrease in glenohumeral external rotation caused by the higher load on the internal rotation muscles during practice, strength and conditioning sessions, and matches [1,14]. The current GIRD paradigm has been defined for pitchers and is based on the retraction of the posterior capsule and the anteriorization of the humeral head [49]. However, the paradigm for the etiology of GIRD does not consider that the increased work of the internal rotator muscles causes its stiffness. Therefore, this stiffness will trigger a restriction in the ER range of movement. When the GERD is maintained for multiple days, the anterior muscles of the shoulder complex will become shortened, and this will bring the humerus to a position of internal rotation, causing the posterior displacement of the humeral head, not anterior displacement as is described for pitchers. The displacement of the humerus to IR will reduce the attachment angle of the posterior rotator cuff, reducing its torque to produce glenohumeral external rotation and, thus, reducing the efficiency of the posterior rotator cuff. Suddenly, the posterior rotator cuff will have to develop more displacement of the humeral head to escape from the subacromial impingement. The increment in the displacement produces increased work, fatigue, and tightness in these muscles. Thus, when this situation persists over time, it will lead to biofeedback that will trigger posterior rotation cuff atrophy, weakness, and shortness, and finally, we will observe an IR restriction [34].

Regarding the testing procedures, we did not use a rolled towel under the elbow of the participants for several reasons. Firstly, the 10° to 15° of divergence between the plane of the humerus and the plane of the scapula was eliminated by gently pressing on the patient’s shoulder throughout the test, sinking the scapula into the foam of the massage table without any discomfort caused to the participants. Secondly, we decided not to use a towel to support the elbow since the pressure maneuver already equalized the planes of movement and fixed the scapula. Furthermore, the use of a towel did not seem to us to be a reliable method, since different arrangements of the towel (more or less folded or rolled) can cause different heights of the humerus, different changes in the degrees between the scapula and the humerus, and differences in the glenohumeral ligament tension; consequently, this could vary the conditions of the evaluation between subjects, which would reduce the reliability between attempts and between tests. Since all of the tests were performed on the same table, by the same experienced researcher, and under the same conditions of pressure on the coracoid process, this aspect added an element of integral reliability to the test.

Most previous studies on the glenohumeral rotational ROM have focused on passive conditions and have used a gyroscope or a goniometer [1,11,16,24,27]. Nevertheless, all tennis strokes are performed quickly and under active conditions. For this reason, we assessed the glenohumeral rotation under active conditions and used another method of evaluation, avoiding the problems reported in previous studies when a single researcher performed the measurements with a goniometer or when two observers were needed to assess one shoulder [1,4,14,27]. It has been demonstrated that changes in positioning among measurements can reduce the reliability of these measurements. Normative values should be established with methods that could be used on non-athlete populations. Nevertheless, most normative values have been established for testing positions using non-functional methodologies [23]. For this reason, we used inertial measurement units (IMUs), which have been demonstrated to be reliable and valid devices to assess the glenohumeral range of motion and more functional movements [28]. We encourage future researchers to assess the glenohumeral ROM with IMU sensors. These devices enhance the ecological validity of these kinds of studies, as the classical goniometer requires more time and has been demonstrated to be less reliable than these sensors [28]. Moreover, only one researcher is needed to assess each athlete to obtain the most accurate normative values. The use of IMUs allows the development of an assessed range of movement without restrictions on the assessment position or conditions, nor the velocity of the observed movement. Nevertheless, further research is needed to assess the glenohumeral rotational motion at different angular velocities [28,29,30].

Another important contribution of the present study is the consideration of GIRDs and GERDs as percentages of deficit between the dominant and non-dominant IR, ER, and TROM. The results showed that, for a pathological GIRD Type II (a percentage of difference between the dominant and non-dominant sides of >10% of the TROM), a classic GIRD had mean values of 16.4 ± 12.8° at 90° of abduction and 14.3 ± 11.1° at 45° under passive conditions, and 16.7 ± 16.9° at 90° of abduction and 16.8 ± 14.1° at 45° under active conditions (Table 4). Additionally, all of the TROMDs were greater than the 5° of difference that previous research has considered as a risk factor for shoulder injuries [14,18,19].

As a practical application, having knowledge about the GERD reference values for experienced tennis players could be important for the design of programs to increase the length of the shortest muscles in the limb, which present the greatest restrictions on external rotation, in order to prevent these restrictions from leading to future discomfort, biomechanical problems, or injuries of the scapulohumeral complex of this side. Another application may be to have data on the normative values for the glenohumeral internal, external, and total rotation, evaluated actively and passively, to compare the specific characteristics of tennis players at levels and ages similar to the sample of the present study. Thus, these data would enable adjustments to strength and conditioning programs to avoid future muscular asymmetries, which are associated with deficits in the internal and external rotation of the glenohumeral joint, both in professional and non-professional tennis players. Moreover, coaches could choose between assessing at 45° or 90° depending on the technical deficits observed in their athletes, thus helping professional athletes avoid overuse problems and enabling non-professional tennis players to guarantee their correct technical development. For example, a proper tennis drive requires a glenohumeral position that optimizes power, precision, and control. This position involves several key biomechanical elements: The shoulder should be slightly abducted, typically between 45° and 90°, to allow a wide range of motion and take advantage of the torso and shoulder muscles for power generation. During the preparation phase, the shoulder should be externally rotated to store elastic energy in the external rotator muscles. This enables a powerful and efficient strike on impact. However, an active or passive GERD could have a negative impact on the preparation phase by reducing the possible power of the stroke. Additionally, at the moment of impact, the shoulder should be in a neutral or slightly internally rotated and adducted position, aligned with the racket’s trajectory. Restrictions in the IR could disrupt the right inertial torque and lead to difficulties in the control and accuracy of the stroke. When executed correctly, the glenohumeral joint will be positioned to maximize force transfer while minimizing the stress on the surrounding tissues. Thus, the early detection of limitations in the range of motion of the glenohumeral joint can help in the design of strength-training programs to prevent biomechanical issues and improve the technical performance of tennis players.

For the time being, the normative reference values provided in the present study may be useful for interpreting GIRDs and GERDs at 45° and 90° of abduction for active and passive conditions. However, further research is needed to provide more data on specific glenohumeral rotation deficits at 45° and 90° under both active and passive conditions, and for different angular velocities. It is essential to understand the technique of the athlete we want to analyze, since each sports technique involves different joint amplitudes and different work angles of the muscles that surround the athlete’s joints. Furthermore, in the same sport, there may be different styles that completely change the biomechanics of the sporting gesture and, consequently, the angle at which the muscles work and the effort that these muscles make or how they will adapt to that effort. Normative values of shoulder rotation at 90° and 45° of abduction under active and passive conditions could be used in clinical and research settings to contextualize individual test values in comparison to age-matched peers.

The present study has several limitations. Firstly, it focused exclusively on asymptomatic tennis players, which restricts the applicability of the findings to individuals with shoulder pathologies or athletes from other sports. The conclusions are based on passive and active external rotation measurements at 45° and 90°, which might not fully represent the dynamic and diverse positions encountered during actual tennis gameplay. Additionally, although this study emphasized the importance of standardized conditions for assessing external rotation, it did not account for potential variations in measurement methods across different evaluators or equipment, potentially impacting the reproducibility.

Furthermore, this study did not examine the long-term effects of rotational deficits or how these measurements might evolve with intervention, limiting its utility for designing injury-prevention strategies. Its focus on the glenohumeral joint overlooked the role of the broader kinetic chain (e.g., scapular stability, core strength, or lower limb involvement), which includes critical components of tennis performance and injury prevention. This research did not address how differences in skill level, training practices, or playing style could influence the range of motion measurements. Moreover, this study relied on static passive and active assessments, potentially underestimating the complexities of the high-velocity and dynamic movements that are typical in gameplay. Finally, this study does not provide details about the sample size or diversity in terms of the gender, age, or hand dominance of the participants, which could limit the generalizability of the results to wider tennis populations. Future research is needed to address these limitations and enhance the robustness and applicability of the findings.

## 5. Conclusions

The results of this study provide evidence on the importance of assessing external rotation under the same conditions, considering the position of the glenohumeral joint at 90°or 45°. In addition, they highlight the importance of determining the external rotation at the angle where more actions are repeated in tennis (45°).

The results suggest that passive measurements of the glenohumeral rotational motion at 90° or 45° showed significantly different results. However, it seems that, when the amplitude is actively performed, there are no significant differences or important effect measurements for either the dominant or non-dominant side at 90° and 45°.

Evaluating GERDs in asymptomatic tennis players could help avoid future biomechanical and GIRD problems. Both GIRDs and GERDs should be considered as a percentage of the athlete’s own deficit in IR or ER, instead of using specific degrees that have been observed in baseball pitchers as a reference.

These data will be useful for comparing tennis players in these specific positions. These data have clinical applications for the rehabilitation of athletes with unilaterally dominant upper extremities and provide a normative database for the full rotational range of motion of the glenohumeral joint in professional tennis players.

## Figures and Tables

**Table 1:** Descriptive characteristics of the participants.

Age (year), mean (SD)	19.1 (2.75)
Gender, *n* female (%)	2
Body mass, mean (SD)	69.7 (10.3)
Shoulder injury history, *n* yes (%)	29 (67.4%)
Shoulder pain during practice, *n* yes (%)	11 (25.5%)
VAS (0–10) of pain, *n* (%)	1 (18%)
3 (45.4%)	
4 (9.09%)	
5 (9.09%)	
6 (9.09%)	
7 (9.09%)	
Shoulder pain during competition, *n* yes (%)	9 (20.9%)
VAS (0–10) of pain, *n* (%)	
4 (44.4%)	
5 (44.4%)	
6 (11.1%)	
Withdrawn from match because of shoulder pain	16 (32.21%)
Dominant right limb, *n* yes (%)	38 (88.37%)
Strength and conditioning (min/week), mean (SD)	110 (11.6)
Tennis practice (hours/week), mean (SD)	9.79 (3.2)
Practices plus matches (hours/week), mean (SD)	17.2 (5.65)
Tennis experience (years), mean (SD)	10.7 (4.4)
Competition experience (years), mean (SD)	6.86 (2.95)
Involved in an injury-prevention program, *n* yes (%)	37 (86.04%)

**Table 2:** Inclusion criteria.

Inclusion Criteria
Must be part of a high-performance tennis club/academy. Must have a minimum of 2 years of experience at this high level. Must not suffer from any ailment or discomfort that would prevent him/her from competing or performing the shoulder rotation range of motion. Must have achieved an elite status and hold national rankings in their respective age categories. Must not be taking medications throughout the study. Must have been free of musculoskeletal injuries over the previous three months.

**Table 3:** Descriptive scores for IR, ER, and TRROM for 90° and 45° of abduction under both active and passive conditions.

	Passive Condition (Supine Test)								Active Condition (Seated Test)							
	Dominant				Non-Dominant				Dominant				Non-Dominant			
Outcome	90°	45°	ES	*p*	90°	45°	ES	*p*	90°	45°	ES	*p*	90°	45°	ES	*p*
IR	68.5	69.9	−0.15	0.31	83.9	99.7	0.18	0.23	74.0	77.7	−0.52	0.001	85.3	90.2	−0.22	0.14
	(12.5)	(15.8)			(15.1)	(12.4)			(9.7)	(20.2)			(20.4)	(20.6)		
ER	108.0	98.3	1.04	0.001 *	111.0	102.0	0.55	0.001 *	105.0	99.1	0.27	0.08	107.0	97.6	0.57	0.001 *
	(10.1)	(11.9)			(13.7)	(16.6)			(16.8)	(23.0)			(22.2)	(24.8)		
TRROM	176.0	168.0	0.58	0.001 *	195.0	184.0	0.57	0.001 *	175.0	177.0	−0.05	0.72	193.3	188.0	0.14	0.33
	(15.5)	(20.4)			(21.6)	(24.3)			(25.0)	(29.3)			(29.6)	(36.3)		

Results are expressed as mean (SD) degrees. The significance level * was set at *p* < 0.05.

**Table 4:** Descriptive scores for three types of GIRDs, GERDs, and TRROMDs at 90° and 45°.

	Passive Condition (Supine Test)		Active Condition (Seated Test)	
Outcome	90°	45°	90°	45°
GIRD (SD)° Min−Max	16.4 (12.8)°	14.3 (11.1)°	16.7 (16.9)°	16.8 (14.1)°
	0−52.6°	0.3–45°	0.3–96.7°	0.3–62.3°
GIRDII (SD)% Min–Max	10.6 (7.3)%	11.4 (8.5)%	10.4 (14.1)%	11.6 (10)%
	0–29.1%	0.2–38.1%	0–38.7%	0-57.5%
GIRD III (SD)% Min–Max	18.5 (12.8)%	16.7 (11.9)%	18.2 (14.7)%	18 (13.5)%
	0–48.1%	0.5–45%	0.4–69.1%	0.3–51.1%
GERD (SD)° Min–Max	10.5 (7.9)° 0–31°	12.0 (11.1)° 0–47.7°	12.1 (8.8)° 0.3–36.3°	15.5 (15.5)° 0.3–82.7°
GERD II (SD)% Min–Max	8.9 (6.5)% 0–27.7 %	11 (9.4) % 0–38 %	10.5 (6.6)% 0.3–26.1%	13.8 (11.3)% 0.4–50.9%
TRROMD (SD)° Min–Max	21.7 (16.4)° 0–64°	21.94 (9.4)° 0–75.3°	22.1 (21.1)° 0–92.6°	23.0 (20.1)° 0–97°

Results are expressed as mean (SD) degrees (°) and percentages (%).

## Data Availability

The data presented in this study are available on request from the corresponding author. The data are not publicly available due to privacy or ethical restrictions.
